# Effect of chlorhexidine varnish on gingival growth in orthodontic patients: a randomized prospective split-mouth study

**DOI:** 10.1590/2177-6709.20.5.066-071.oar

**Published:** 2015

**Authors:** Henrique Pretti, Gabriella Lopes de Rezende Barbosa, Elizabeth Maria Bastos Lages, Alfonso Gala-García, Claudia Silami de Magalhães, Allyson Nogueira Moreira

**Affiliations:** 1 PhD resident in Oral Radiology, Universidade Estadual de Campinas (UNICAMP), Campinas, São Paulo, Brazil; 2 Adjunct professor, Universidade Federal de Minas Gerais (UFMG), Belo Horizonte, Minas Gerais, Brazil; 3 Associate professor, Universidade Federal de Minas Gerais (UFMG), Department of Restorative Dentistry, Belo Horizonte, Minas Gerais, Brazil

**Keywords:** Chlorhexidine, Gingival diseases, Gingivitis, Orthodontic appliances, Tooth Crown

## Abstract

**Introduction:**

Fixed orthodontic appliances patients suffer limitations on the effective control of biofilm by mechanical methods, bringing the need of a coadjutant in the control of inflammation and oral health improvement.

**Objective:**

The aim of this prospective split-mouth blind study was to analyze the effect of a 40% chlorhexidine (CHX) varnish on gingival growth of patients with orthodontic fixed appliances. Methods: Healthy teenage patients with fixed orthodontic appliances and increased gingival volume were recruited (n = 30). Each individual was his own control, having in the maxilla one control side and one treatment side. An application of varnishes occurred on the vestibular area of the upper premolars and first molar crowns, on the control side (placebo varnish) and on the experimental side (EC40^(r)^ Biodentic CHX varnish). The varnishes and sides were randomly chosen and its identification and group was kept by a third party observer and it was not revealed to the researchers and participants until the end of study. In order to establish a baseline registration, digital photographs were taken by a trained photographer before varnish application at baseline (T_0_), as well as 14 days (T_14_) and 56 days (T_56_) after the application. The gingival volume was calculated indirectly using the vestibular areas (mm^2^) of the upper second premolars' clinical crowns by RapidSketch^(r)^ software, at all study times. The data were analyzed using ANOVA and the Turkey-Krammer test.

**Results:**

It was observed, in the final sample of 30 individuals, that at T_0_, the control and treatment groups were similar. At T_14_ and T_56_, a progressive reduction of the clinical crown area was seen in the control group, and an increase in the average area was detected in the experimental group (*p* < 0,05).

**Conclusions:**

The use of 40% CHX varnish decreases the gingival overgrowth in patients undergoing orthodontic treatment. Further studies are necessary to set the action time and frequency of application.

## Abstract (pt)

**Introdução:**

pacientes com aparelhos ortodônticos fixos sofrem limitações no controle efetivo de biofilme por métodos mecânicos, trazendo a necessidade de um coadjuvante no controle na inflamação e melhora na saúde bucal.

**Objetivo:**

esse estudo cruzado prospectivo randomizado teve como objetivo analisar o efeito do verniz de clorexidina (CHX) a 40% no crescimento gengival de pacientes com aparelhos ortodônticos fixos.

**Métodos:**

indivíduos adolescentes com aparelhos ortodônticos fixos e aumento de volume gengival foram recrutados para a pesquisa (n = 30). Cada participante atuou como seu próprio controle, tendo, na maxila, um lado controle e um tratamento. No lado controle, aplicou-se verniz placebo e no lado experimental, o verniz EC40^(r)^ Biodentic CHX, ambos na face vestibular das coroas dos pré-molares e primeiro molar superiores. Os vernizes e lados foram escolhidos de forma aleatória e a identificação deles e a que grupo pertenciam foi mantida por um terceiro observador, não sendo revelada aos pesquisadores nem aos participantes até o final do estudo. Fotografias digitais foram tiradas por um fotógrafo treinado, antes da aplicação do verniz no tempo inicial (T_0_), bem como 14 dias (T_14_) e 56 dias (T_56_) após a aplicação. O volume gengival foi calculado indiretamente, por meio das áreas vestibulares (mm^2^) das coroas dos segundos pré-molares superiores, com o *software *RapidSketch^(r)^, em todos os tempos de estudo. Os dados foram analisados usando ANOVA e teste de Turkey-Krammer.

**Resultados:**

na amostra final de 30 indivíduos, observou-se que, em T_0_, os grupos controle e tratamento foram semelhantes. Já em T_14_ e T_56_, foi observada uma progressiva redução na área da coroa clínica no grupo controle, e um aumento na área média do grupo experimental (*p *< 0,05).

**Conclusão:**

o uso do verniz de CHX a 40% diminui o excessivo crescimento gengival em pacientes sob tratamento ortodôntico. Estudos futuros são necessários para se determinar o tempo de ação e a frequência de aplicação.

## INTRODUCTION

During orthodontic treatment, patients wearing fixed appliances have additional plaque buildup, as well as increased stimulated salivary flow and inflammatory reaction of gingival tissues.^1^^,^ 2 Plaque increases around bands, orthodontic adhesives and brackets; the composition of the oral flora changes; and cleaning becomes more difficult for the patient.^3^ Brackets, bands and other accessories hinder cleaning, which can cause enamel demineralization, dental cavities and gingival swelling.^4^ The appearance of carious lesions during orthodontic treatment can be explained mainly by inadequate plaque control due to fixed appliances use.^5^

Orthodontic patients need to implement an oral hygiene preventive program and pay closer attention to oral hygiene^6^^,^ 7 which is particularly difficult to maintain when bands, wires and other accessories are present. In this sense, the effective control of dental plaque by mechanical methods suffer some limitations in fixed orthodontic appliances patients.^8^^,^ 9

With the implementation and application of oral health preventive programs for patients wearing fixed appliances, patients should be motivated in order to improve their oral health and, under clinical supervision, be more successful in eliminating plaque and inflammatory symptoms.^10^

Thus, the important role of chemical agents used to improve oral health should be considered. The use of these substances (mouthrinses or dentifrices) might help reduce biofilm buildup on soft tissue surfaces in the oral cavity, potentially delaying plaque accumulation over teeth. However, antimicrobial agents, such as essential oil mouthrinses and dentifrices containing triclosan/copolymer, might affect the subgingival microbiota by disrupting contiguous supragingival plaque. Similarly, the use of a dentifrice containing triclosan/copolymer might prevent the progression of attachment loss in adolescents with a high risk of developing early periodontitis and might prevent further loss of attachment in patients with a history of periodontitis, particularly in the absence of a supportive periodontal therapy that includes subgingival debridement.^11^

The use of chlorhexidine (CHX) as an agent that prevents caries and gingival disease is common. The mechanism of action of chlorhexidine against microorganisms is explained by the cationic ligation to the negatively charged cell walls, which destabilizes osmotic balance, causing precipitation or coagulation of the cytoplasmic content that kills the cells. CHX is considered the gold standard of antimicrobial mouthrinses in Dentistry.^12^

CHX use has several advantages, such as its anti-bacterial spectrum that covers gram-positive and gram-negative bacteria, fungi and yeasts to a lesser extent. Also, its substantivity, the ability of an agent to be retained in particular surroundings, is due to its ability to bind to carboxyl groups of the mucin that covers the oral mucus and to be steadily released from these areas in an active form, displaced by the calcium ions segregated by salivary glands. The use of CHX also has disadvantages because it is not a virucide, nor it is effective against acid-alcohol resistant bacilli. Furthermore, its taste is unpleasant and staining of teeth occurs when used in the form of mouthwashes in the long term.^13^

The vehicles most often used to administer CHX are mouthrinses (at concentrations of 0.12% and 0.2%), aerosols (0.12% and 0.2%), gels (0.12% and 1%) and varnishes.^13^^,^ 14

Varnishes have been developed over the past decade. They are the most effective form of CHX professional application, as they are easy to apply, do not require patients to cooperate, and although they have an unpleasant flavor, they do not cause discoloration.^15^ Initially, CHX varnish was tested for the prevention of caries in high-risk populations and was implemented as a treatment strategy for chronic periodontitis.^16^ Currently, three CHX varnishes are manufactured: Clorzoin^(r)^, EC40^(r)^ and Cervitec^(r)^. Chlorhexidine composition and concentration of EC40^(r)^ varnish are 40% chlorhexidine, 36% sandarac and 24% ethanol. Indeed, numerous applications of EC40^(r)^ on the tooth surface create a reservoir of CHX, thereby suppressing microorganisms of supragingival plaque, and, thus, lowering their pathogenic potential.

The use of varnish avoids the undesirable effects of CHX, such as altered taste, extrinsic staining of the enamel and the need for patient's cooperation. Low bacterial activity, maintenance of oral flora balance, excellent absorption by the enamel surface and good tolerance by patients are expected.^15^^,^ 16

Therefore, treatment strategies using chlorhexidine varnish to prevent early microbial recolonization ultimately ensure the best chance for clinical improvements. EC40^(r)^ is notably a highly concentrated, easy-to-use CHX varnish which can be injected into the periodontal pocket. To date, this varnish has been mainly tested for the prevention of caries in high-risk populations.^16^^,^ 17

The hypothesis that CHX would have beneficial effects for orthodontic patients was raised because of the need for adjuvant therapies for these patients, as well as because of the proven effects of this varnish in improving cases of chronic gingivitis, plaque buildup and bleeding levels.^13^

In this study, we assessed the effect of a 40% CHX varnish on gingival growth of patients undergoing orthodontic treatment, analyzed by a computerized area evaluation of teeth crowns.

## MATERIAL AND METHODS

This study was previously approved by Universidade Federal de Minas Gerais Institutional Review Board (protocol #114/8). A total of 30 participants (14 males and 16 females) aged between 12 and 17 years old who had been assisted at the Department of Orthodontics of the same university were included in the study after they were informed, along with their legal guardians, of the research purpose. They also signed an informed consent form. The number of participants was defined by convenience sample.

The following inclusion criteria were applied: patients wearing orthodontic fixed appliances over six months and presenting gingival overgrowth Grade 2^18^^,^ 19 in maxillary premolars and molars, as diagnosed by a previously trained examiner (Kappa = 0.92).

Subjects who had any of the following factors, which could influence gingival growth, were excluded: antibiotic therapy for the past three months or during the study period; use of anticoagulants, immunosuppressants, calcium channel blockers, or other medication that causes gingival swelling; pregnant and lactating women; signs of candidiasis; previous use of chlorhexidine as toothpaste or mouthwash for at least 30 days; report of allergic reactions to any component of the varnish; smokers; and patients who had undergone periodontal surgery or extraction of adjacent studied teeth in the past four months.

Oral and written guidelines of adequate oral hygiene with modified Bass brushing technique and flossing were given after the placement of fixed orthodontic appliances. All appliances placed on patients were made of the same material from the same brand. Research subjects were requested to follow the same oral hygiene standard before and during the experiment. The CHX varnish used was EC40^(r)^ varnish (Biodent, Arnhem, Netherlands) which consists of 40% CHX, 36% sandarac and 24% ethanol; whereas the placebo varnish contained 60% sandarac and 40% ethanol (Fórmula & Ação, São Paulo, Brazil). Varnish was applied only once on the buccal surface of maxillary premolars and first molar crowns on the right and left sides. One side was randomly selected as control and received placebo varnish, while the other side, the experimental one, received 40% CHX varnish. The key that identified the varnishes and which group they belonged to was kept by a third party and was not revealed to the researchers until the study was over.

The application procedure was performed by the same dentist using dental equipment and following the manufacturer's instructions. At first, the teeth were cleaned with a toothbrush for 2-3 minutes, then, they were isolated from saliva with cotton rolls and dried with compressed air. Subsequently, a thin coat of varnish was applied on the buccal surface of teeth around orthodontic brackets and along the gingival margin using a cartridge syringe fitted with a blunt needle. Excess varnish was removed after seven minutes. Participants were instructed not to eat or drink for three hours and not to clean their teeth until the following day. Patients were not informed which varnished was used on each maxillary side.

A trained photographer used a digital camera (Canon Rebel) with 100 mm macro ring flash to take intraoral photographs of the right and left sides of the patient before varnish application (T_0_), 14 days after application (T_14_), and 56 days after application (T_56_). All photographs were taken by the same photographer, standardizing the position of the occlusal plane parallel to the floor and the premolar region with equivalent angulation (90 degrees).

**Figure 1:**
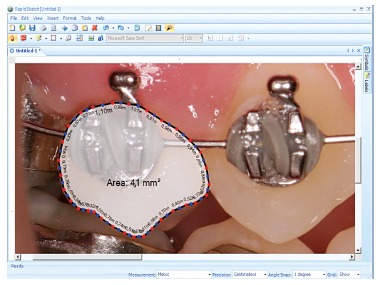
- Rapid Sketch^(r)^ software used to measure second premolar crown area.

By means of Rapid Sketch^(r)^ software v. 2.4 (Utilant, Buffalo, NY, USA), the digital photographs were analyzed and the buccal areas (mm^2^) of second premolars clinical crowns were measured using the software tools that allow the surface of interest to be determined and calculated (Fig 1). Gingival growth was also calculated indirectly by the software, as proposed by Rodrigues et al.^20 ^This procedure was performed for all photographs at all study times (T_0_, T_14_ and T_56_). Data were submitted to one-way ANOVA and Tukey-Kramer test (a = 5%).

## RESULTS

Thirty patients aged between 12 and 17 years old completed the study. The distribution of sample sites was equal in both groups (control side and treatment side), and the assessment of clinical data of sample sites was performed at three different time intervals: 0, 14, and 56 days after application.

Split-plot ANOVA revealed statistically significant differences between treatment modalities and study times. The time-treatment interaction was statistically significant (*p* < 0.05). Tukey-Kramer test compared the effects of time and treatment approaches.

The increase in clinical crown area indirectly represents gingival volume decrease. When the different study periods were compared, at T_0_, there was no difference between areas in control and treatment groups (*p* > 0.05). However, at T_14_ and T_56_, the means of areas in the treatment group were statistically higher than the control group (*p* < 0.05) (Table 1).

Regarding the comparison of study periods, in the control group, the mean area did not differ between T_0_ and T_14_, but was significantly smaller at T_56_ (*p* < 0.05). Additionally, in the treatment group, there was a significant increase in area from T_0_ to T_56_, being progressive during the period of study (*p* < 0.05) (Table 1).

**Figure 2:**
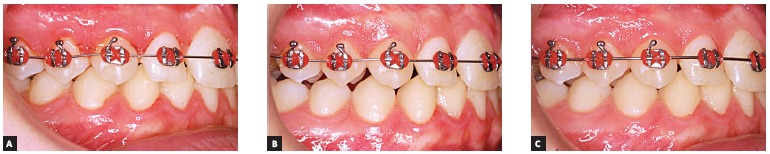
- Control side: intraoral photographs at T_0_ (A), T_14_ (B) and T_56_ (C).

**Figure 3:**
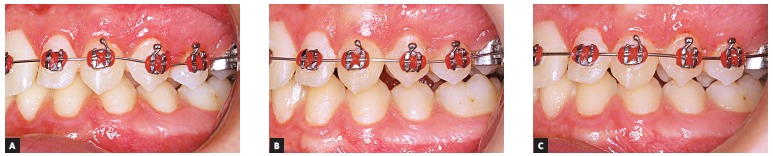
- Treatment side: intraoral photographs at T_0_ (A), T_14_ (B) and T_56_ (C).

The increase in tooth area in the treatment group and decrease in the control group can also be observed clinically, as illustrated in Figures 2 and 3.

**Table 1:** - Buccal areas (in mm

**Time**	**Group**	
	**Control**	**Treatment**
T_0_	4.540 ± 0.802 ^a,A^	4.537 ± 0.799 ^a,A^
T_14_	4.473 ± 0.831 ^a,A^	4.717 ± 0.829 ^b,B^
T_56_	4.367 ± 0.792 ^a,B^	4.940 ± 0.865 ^b,C^

Means followed by different letters (small letters in lines and capital letters in columns) are significantly different according to Tukey-Kramer test (*p* < 0.05).

## DISCUSSION

This is a prospective clinical split-mouth study designed to assess the effect of a 40% CHX varnish (EC40^(r)^ Biodentic) on the gingival growth of orthodontic patients. The split-mouth design is considered an adequate study design that has been used in different clinical areas.^21^^,^ 22 For its application, more than one site must be affected by disease in the mouth, therefore, in this study, only patients with both left and right maxillary affected sides were chosen. In addition, the similarity between control and treatment groups at T_0_shows that both groups were homogeneous at the beginning of the study and each subject adequately worked as its own control.

Based on the properties of the available substances and the clinically proven results, CHX, in comparison to other products, is considered the gold standard in inhibiting plaque formation and gingival overgrowth.^12^ However, there are some side effects that result from its continuous use as a mouthwash and toothpaste. Potential adverse side effects most common during CHX treatment are as follows: temporary palate disorders, tooth staining, or unpleasant taste;^23^but these disadvantages were controlled in the present study by using CHX in the dosage form of varnish.

In this study, the proposed method allows gingival alterations to be measured by modification of the clinical crown area of teeth. By means of computerized analysis, which has been previously reported, a numeric area variation was provided, enabling quantitative assessment and a more accurate statistical analysis.^20^ Area measurement by means of computerized image analysis of digital photographs has been studied and proven to be effective.^24^ However, photographs should be well-taken to avoid focus and angle errors by the operator. Thus, a well-trained photographer is necessary for standardization of photographs.^25^

A progressive increase was observed in the clinical crown area of second premolars 14 and 56 days after application of 40% chlorhexidine varnish. These results demonstrate the effects of CHX varnish that acts against microorganisms responsible for gingival overgrowth.^12^Moreover, this antimicrobial agent provides an additional feature, in particular, its sustained-release and substantivity property, which decreases the level of microorganisms in patients with fixed appliances.^26^^,^ 27 Other studies report that EC40^(r)^ has a good performance, decreasing microorganisms in gingival plaque up to six months after application,^28^^,^ 29 which can explain the positive results observed on patients under treatment even 56 days after (T_56_) CHX application. Microorganism control must be performed as periodontal complications arise during adolescent orthodontic therapy, and gingival inflammation is associated with the presence of periodontal pathogens, both supragingivally and subgingivally.^30^

Conversely, in the control group, there was no significant increase in gingival volume during the period of two weeks (T_14_), probably because crown polishing was performed before CHX application (T_0_). However, after 56 days (T_56_), the gingival inflammatory process could be demonstrated by statistically significant increase in gingival volume.

Since CHX varnish proved positive as adjunct during orthodontic treatment, it is necessary to quantify application time and frequency. It is also important to determine the ability of 40% CHX varnish to maintain reduced gingival hyperplasia by means of long-term longitudinal studies, since a progressive reduction in gingival growth could be observed along the 56 days of the study. Further studies are needed using larger sample sizes, as well as researches assessing the effect of CHX varnishes in combination with mechanical plaque control, since these are the limitations of the research.

## CONCLUSION

The use of 40% CHX varnish promoted a progressive increase in clinical crown area at 14 and 56 days after application. CHX varnish proved effective against gingival overgrowth in patients undergoing orthodontic treatment. Further studies are necessary to set the action time and frequency of application of the substance.
